# Implicit processing during change blindness revealed with mouse-contingent and gaze-contingent displays

**DOI:** 10.3758/s13414-017-1468-5

**Published:** 2018-01-23

**Authors:** Andrey Chetverikov, Maria Kuvaldina, W. Joseph MacInnes, Ómar I. Jóhannesson, Árni Kristjánsson

**Affiliations:** 1 The Icelandic Vision Laboratory, University of Iceland, Reykjavik, Iceland; 2 Cognitive Research Lab, Russian Academy of National Economy and Public Administration, Moscow, Russia; 3 Department of Psychology, Saint Petersburg State University, Saint Petersburg, Russia; 4 School of Psychology, National Research University: Higher School of Economics, Moscow, Russia

**Keywords:** Change blindness, Eye movements, Attention, Gaze-contingent, Mouse-contingent, Pupil size

## Abstract

People often miss salient events that occur right in front of them. This phenomenon, known as change blindness, reveals the limits of visual awareness. Here, we investigate the role of implicit processing in change blindness using an approach that allows partial dissociation of covert and overt attention. Traditional gaze-contingent paradigms adapt the display in real time according to current gaze position. We compare such a paradigm with a newly designed mouse-contingent paradigm where the visual display changes according to the real-time location of a user-controlled mouse cursor, effectively allowing comparison of change detection with mainly overt attention (gaze-contingent display; Experiment 2) and untethered overt and covert attention (mouse-contingent display; Experiment 1). We investigate implicit indices of target detection during change blindness in eye movement and behavioral data, and test whether affective devaluation of unnoticed targets may contribute to change blindness. The results show that unnoticed targets are processed implicitly, but that the processing is shallower than if the target is consciously detected. Additionally, the partial untethering of covert attention with the mouse-contingent display changes the pattern of search and leads to faster detection of the changing target. Finally, although it remains possible that the deployment of covert attention is linked to implicit processing, the results fall short of establishing a direct connection.

**Electronic supplementary material:**

The online version of this article (10.3758/s13414-017-1468-5) contains supplementary material, which is available to authorized users.

If attention is otherwise occupied, people often miss large changes to unattended parts of the environment—a phenomenon known as “change blindness”. Despite 25 years of research since the seminal demonstration by O’Regan (1992), the mechanisms behind this phenomenon are not clearly understood. While initial accounts of change blindness assumed no detection or storage of the missed transient signal (O’Regan, 1992; Rensink, 2000), others argued for either a lack of comparison between the two presented signals (Beck & Levin, 2003; Mitroff, Simons, & Levin, 2004) or that representations lack point-by-point detail leading to complications during comparisons (Henderson & Hollingworth, 2003). The latter accounts suggest that information necessary for change detection is implicitly perceived and stored but not always used. This lack of comparison or impoverished representations may be caused by different mechanisms, with one of the possible candidates being a dissociation of overt and covert attention. We aimed to study change detection and implicit processing of undetected changes using novel gaze-contingent and mouse-contingent displays that enable a comparison of tethered and untethered overt and covert attention.

Behavioral experiments of change detection usually show that unnoticed changes are processed to some extent. Semi-explicit responses (mindsight; Rensink, 2004) or forced-choice procedures (Fernandez-Duque & Thornton, 2000, 2003; Laloyaux, Destrebecqz, & Cleeremans, 2006; Mitroff et al., 2004) argue for above-chance registration, localization, and identification of unidentified changes. Reaction time data show that correct selections of missed changing targets elicit longer responses (Koivisto & Revonsuo, 2003; Williams & Simons, 2000) than unchanged items and shorter responses than incorrect selections (Fernandez-Duque & Thornton, 2000, Experiment 1). Priming effects have also been found for prechange and postchange items in comparison with novel items even if the change was not noticed (Caudek & Domini, 2013; Silverman & Mack, 2006; Yeh & Yang, 2009). Similarly, analyses of pupil dilation indicate that even when the change is not detected, processing of changing items involves more attentional engagement than processing of distractors (Vachon, Vallieres, Jones, & Tremblay, 2012). Both behavioral and eye-movement data, therefore, show that unnoticed changing targets are processed to a certain extent. A pertinent question is what determines whether observers miss the change.

Several authors have argued that the answer to this question reflects the work of the attention system (Rensink, O’Regan, & Clark, 1997; Scholl, 2000; Simons & Ambinder, 2005; Tse, 2004). Eye-tracking studies show that overt attention shifts make it easier to find a change than when eye-movements are restricted (Hollingworth, Schrock, & Henderson, 2001) and fixations close to a changing target item are good predictors of finding a change (Henderson & Hollingworth, 1999; Hollingworth et al., 2001; Vachon et al., 2012). However, even fixations on the target and direct overt attention do not guarantee change identification (Caplovitz, Fendrich, & Hughes, 2008; Fudali-Czyz, Francuz, & Augustynowicz, 2014; O’Regan, Deubel, Clark, & Rensink, 2000; Simons & Levin, 1998; T. J. Smith, Lamont, & Henderson, 2012; Triesch, Ballard, Hayhoe, & Sullivan, 2003). This effect was coined “attentive blank stares” and was seen on around 40% of trials (Caplovitz et al., 2008; O’Regan et al., 2000). “Attentive blank stares” were later connected to the amplitude of the lambda response in fixation-related brain potentials that was interpreted as reflecting insufficient attentional processing during encoding (Fudali-Czyz et al., 2014).

## Goals of the present study

The current study had two goals. First, we aimed to obtain a detailed description of implicit target processing available from eye movements preceding “attentive blank stares.” Previous studies have revealed effects of fixation location and pupil size at the moment of change on change detection probability (Henderson & Hollingworth, 1999; Hollingworth et al., 2001; Vachon et al., 2012). In these studies, the analysis of eye movements was limited to a comparison of trials where the change was found, with change blindness trials. However, this comparison on its own does not suffice, as it might reflect processing of a target after the change was detected. To overcome this limitation, we compared eye movements on change blindness trials with catch trials where no change was introduced. Furthermore, the characteristics of eye movements before the crucial moment when observers fixate the change location and either detect the change or not, remain to be studied. On the one hand, it is possible that before this moment, the target is not processed at all and the attentive blank stare is likely to happen on the first fixation on the target. On the other, the target may be analyzed to some extent, but its processing is the same as of any other stimuli. Finally, it is possible that the changing target is insufficiently processed even before the attentive blank stare. We therefore analyze not only eye movements at the moment of change, as in previous studies, but rather during the whole period preceding the attentive blank stare.

Second, we aimed to investigate one of the possible reasons for “attentive blank stares,” namely, the dissociation between overt and covert attention. Overt attention can be measured directly as the focus of gaze. However, covert attention can be disconnected from overt attention (Belopolsky & Theeuwes, 2009; Hunt & Kingstone, 2003; Posner, 1980; Walter, Quigley, Andersen, & Mueller, 2012), and this could explain the lack of attentional engagement at the locus of fixation. The role of covert attention in change detection has been highlighted in previous studies. For example, Scholl (2000), and D. T. Smith and Schenk (2008) showed that presenting visual precues known to produce covert shifts of attention, facilitates change detection when the precues are shown at the change location. In contrast, if the precued location does not contain the change, covert attention shifts would be detrimental to change detection. We reasoned that if we could increase the association of overt and covert attention procedurally (“tether” them together in a gaze-contingent display), then “attentive blank stares” will be less frequent.

## Study design

We conducted two experiments using a novel mouse-contingent and gaze-contingent change blindness (CB) paradigm. Traditional gaze-contingent displays monitor the gaze position of participants and adjust the visible display in real time to show only a small part of the scene centered on the current gaze position. The gaze-contingent paradigm provides an opportunity to control the perceptual span and the focus of overt attention (McConkie & Rayner, 1975; Parkhurst & Niebur, 2002; Reingold, Loschky, McConkie, & Stampe, 2003). However, if the visible area is small, this procedure limits not only covert attention but also implicit processing of peripheral unattended stimuli. To overcome this limitation in our gaze-contingent display, we used a larger visible area with an additional modification to the usual procedure. If observers fixated at one location for some time the visual area around fixation started to shrink so that observers had to shift their gaze to a new position for a less limited view of the display. In this way, we could limit the participant’s ability to use covert attention while keeping the visual field size suitable for implicit processing.

While the gaze-contingent procedure separates foveal from parafoveal input, it also ties the visible area to overt attention. It is difficult for observers to use their covert attention separately from overt attention when everything outside the overtly attended region is reduced or hidden. Our mouse-contingent display, however, allows untethering of covert and overt attention while equating the visible area by tying it to mouse movements controlled by the participant, independently of gaze position. It is usually assumed that mouse-contingent displays are slower to update than gaze-contingent displays are, and mouse moves follow visual directions with a lag of about 70 ms (Reingold et al., 2003). However, it has also been shown that the eyes can monitor the progress of a motor movement (B. A. Smith, Ho, Ark, & Zhai, 2000), motor movements can precede target fixations in predictable contexts (Bieg, Chuang, Fleming, Reiterer, & Bülthoff, 2010), and visual attention can be allocated in parallel (or synergistically) to saccade and reaching targets (Jonikaitis & Deubel, 2011). Mouse-contingent displays therefore allow different patterns of hand–eye coordination but do not restrict covert attention to the same extent as gaze-contingent displays. We predicted that the gaze-contingent “tethered” paradigm would result in an increase in change detection times (because observers would be less able to use covert attention to look for changes), but “attentive blank stares” would decrease (because overt and covert attention will be less likely to be dissociated) in comparison to the mouse-contingent “untethered” condition. That is, observers in the gaze-contingent experiment would take longer to find the change with gaze-contingent displays, but they would miss fewer changes.

Gaze-contingent paradigms with displays limited to specific areas are rare in change blindness research.^1^ We found only two studies (Cañal-Bruland, Lotz, Hagemann, Schorer, & Strauss, 2011; Reingold, Charness, Pomplun, & Stampe, 2001) that both utilized this procedure to estimate the effect of chess and soccer expertise on change blindness. To our best knowledge, neither investigated the role of nonexplicit detection in this task. We also did not find any change blindness studies that utilized mouse-contingent displays. We believe that comparing indices of implicit processing in gaze-contingent and mouse-contingent displays is especially important since attention might be required for implicit processing (Thomas & Lleras, 2009; Turk-Browne, Jungé, & Scholl, 2005). We therefore specifically tested for interactions between attention and implicit processing.

We compared change blindness (CB) trials where the target was found (“found target”; FT trials) and “catch” trials (where there is no changing target), allowing us to distinguish between different stages of change detection. We reason that, first, the difference between CB and catch trials may reveal indicators of implicit processing of changes that does not reach awareness, and, second, that differences between CB and FT trials could indicate later, possibly explicit, processing necessary for conscious awareness of the change (see, e.g., Koivisto & Revonsuo, 2003, for similar comparisons).

Finally, we recently reported that the longer people look at a given stimulus without successfully identifying it in a visual search task, the more negative is their liking rating of that stimulus (Chetverikov, Jóhannesson, & Kristjánsson, 2015), consistent with a general framework linking perception and affective processing (Chetverikov & Kristjánsson, 2016). Mild negative valence (without strong arousal) might, in turn, lead to avoidance of the nonidentified stimulus, decreasing the fixation rate and gaze duration at the nonidentified stimulus (e.g., Simola, Le Fevre, Torniainen, & Baccino, 2015), further decreasing the chances of successful identification. We therefore ask whether such recursive negative feedback can explain failures to notice changes. We test the proposal that negative affect induced by not noticing the target may cause observers to be even less likely than otherwise to notice the change. We used a liking procedure that tests for the role of negative affect and dissociates liking from simple choice biases (Chetverikov & Kristjánsson, 2015).

## Method

### Procedure

**Fig. 1:**
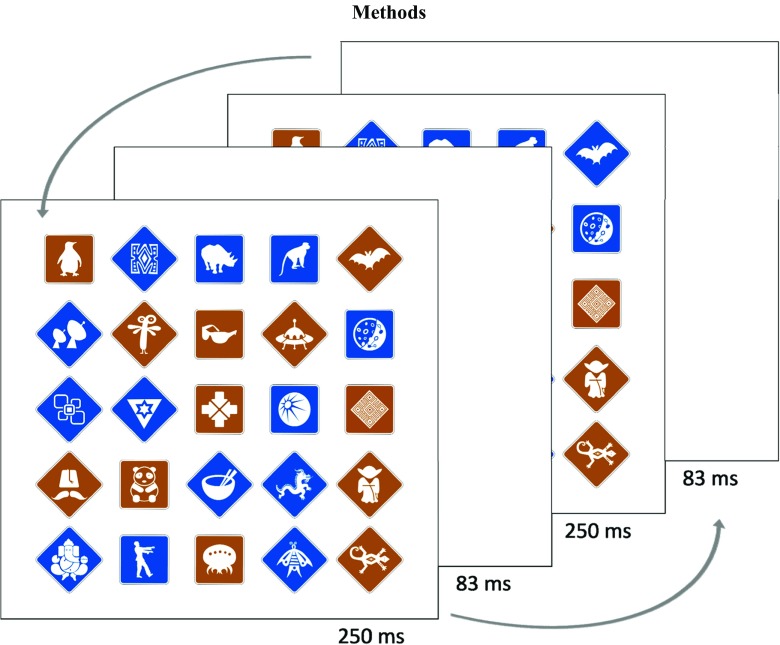
Example of the stimulus matrix and the trial loop. Changed and non-changed versions of the stimulus matrix were shown for 250 ms separated by a 83 ms blank screen. The presentation was repeated until observers found the target or until cursor or gaze remained at target position long enough for the presentation of both versions of the target (see details in text). In this example, the color of the stimulus in the top right corner changes. Part of the stimulus matrix was occluded by the gaze-contingent or mouse-contingent mask not shown here (see Fig. S1 and video in the Supplement).


On each trial, observers were presented with a 5 × 5 matrix of stimuli, with 3.75° of visual angle between their centers. In total, 64 artificial “traffic signs” were used, each of which could be placed on a square (2.5° × 2.5°) or diamond (square rotated 45°) with a blue (RGB: [0, 79, 162] on 0 to 255 range) or brown (RGB: [162, 84, 0]) background (see Fig. 1). The symbol, color, and orientation of each stimulus were chosen randomly for each trial (each symbol was presented only once on each trial). The stimuli were presented for 250 ms (15 frames at 60 Hz), followed by a blank screen for 83 ms (five frames at 60 Hz), followed again by the stimuli, but with one of them changed^2^ (250 ms), and another blank screen (83 ms). This cycle was repeated for the duration of the trial. Trials were separated by a 1,000-ms fixation period. In total, there were 75 change trials (25 positions, three types of changes: color, rotation, symbol on the traffic sign) and 25 catch trials without any changes. Change position was counterbalanced for each change type.

ESM 1(MP4 25486 kb)


The visible area for all trials was restricted and contingent on either mouse position (Experiment 1) or gaze (Experiment 2). This circular area (21.7° in diameter) was defined by a Gaussian (*SD* = 3.61°) mask, transparent at the center, with visibility decreasing in the periphery as a function of the standard deviation. When the cursor (or gaze) stayed within same region (defined as a circle with 1.88° radius) for more than 50 frames (830 ms), the visible region started to shrink by 1% per frame. This forced observers to use the mouse or gaze to investigate the screen instead of simply placing the cursor or gaze at the center. The parameters of the mask were chosen so that when the visible area was centered and had maximal size, the stimuli at the edge of the matrix were partly visible (see Fig. S1 in the Supplement). Postexperimental analyses showed that the mask had the maximal size 84% of the time (95% CI [82, 87]) and shrank on average by 13% (95% CI [12, 14]) when it was not of maximal size in Experiment 1, and 84% of the time (95% CI [82, 86]) with an average shrinkage of 21% (95% CI [17, 24]) in Experiment 2.

The trial ended when one of two conditions was met. First, observers were to indicate that the target was found by clicking on it with the left mouse button (Experiment 1) or just clicking the left mouse button (Experiment 2). The mouse cursor was not visible in Experiment 2 during the search. Otherwise, observers would have to move the cursor to the target when it was found (since the cursor was not needed otherwise, it could be anywhere), which would inevitably add noise to the measurements of search time. Response accuracy was determined by the item observers clicked on (Experiment 1) or by the item observers looked at when they clicked (Experiment 2; note that with this procedure the number of false alarms was identical between the experiments; see Table S1 in the Supplement). The trial also ended if the cursor (Experiment 1) or gaze (Experiment 2) remained 2.5° or less away from the target long enough for both target versions to be presented (for at least one frame) followed by a mouse movement or gaze leaving the target area. In this case, a liking procedure was initiated. Observers were asked to choose their most or least preferred object (“Which one do you like most?” or “Which one do you like least?”). Possible choices were the change target and two distractors closest to the mouse or gaze position that had appeared within an imaginary circle of 3.30° radius around the target. The target was shown with the properties last presented during the search. To counterbalance, observers were asked to choose the least preferred object on half the trials and the most preferred object on the other half. When observers chose one of the stimuli by left-clicking it, it disappeared, and they had to select among the two remaining objects. Finally, they clicked on the last remaining object to begin the next trial. If observers noticed the change without clicking on it in time, they were instructed to right-click it during the liking procedure.

Catch trials were identical to the other trials, except that “targets” here did not change—they were randomly selected distractors (position counterbalanced). That is, on each trial a single stimulus was chosen to be a pseudo target and was treated exactly like a real target to determine if the liking procedure should be initiated. Thus, catch trials ended if observers erroneously indicated that they had found a change or if the cursor (or gaze) stayed close to the pseudo target long enough to trigger the liking procedure, as described above.

### Materials and apparatus

In Experiment 1, the stimuli were presented on a 19-in. Acer V193 display (1280 × 1024 px) with an SMI RED-m 60 Hz eye tracker attached to the bottom of the display. Observers were seated approximately 60 cm from the screen. The experiment was run using PsychoPy (Peirce, 2007, 2009). In Experiment 2, observer’s heads were stabilized with a chin rest and headrest. Stimuli were displayed on a 19-in. Hansol 920D CRT screen (1024 × 768 px). Viewing distance was 56 cm. Mouse position was sampled at 60 Hz. A monocular 250-Hz eye tracker from Cambridge Research Systems (2006) monitored eye position (see Jóhannesson, Ásgeirsson, & Kristjánsson, 2012). The experimental software was written in MATLAB using Psychtoolbox (Brainard, 1997; Kleiner, Brainard, & Pelli, 2007; Pelli, 1997). Fixations were calculated based on the IV-T algorithm (Salvucci & Goldberg, 2000) with a 40 degree per second threshold and polynomial smoothing of gaze position to reduce noise. Although two different eye trackers were used, the same algorithm (I-VT) was adopted for both. Polynomial smoothing of samples was used to reduce noise and fixation detection parameters were set to motion 40 degrees per second or less plus a 50 ms minimum duration.

### Participants

Thirty-two observers (18 women, 18–27 years old, age *Mdn* = 21 years) took part in Experiment 1 (mouse-contingent display) at Saint Petersburg State University. Twenty-four observers (18 women, 18–51 years old, age *Mdn* = 23 years) took part in Experiment 2 (gaze-contingent display) at the University of Iceland. Observers were not paid for participation. All reported normal or corrected-to-normal visual acuity. The experiments were approved by the ethics committees of the corresponding universities.

## Results

Given the number of comparisons and dependent variables, we provide a summary of the results in Tables 1 and 2, followed by a detailed description for each variable. Table 1 provides a summary of the differences between the two experiments that highlight the role of covert attention. Table 2 provides a summary of comparisons between change blindness (CB) trials and catch trials that highlight the role of implicit processing. A comparison of CB or catch trials with “found target” (FT) trials is described in the following paragraphs but is not included in the summary tables.

**Table 1:** Decoupling attention: tethered attention (Experiment 2 [E2], eye-contingent display) versus untethered (Experiment 1 [E1], mouse-contingent display)

Measure	Result	Interpretation
Change detection times and accuracy	E1 faster than E2	Untethered covert and overt attention yields a performance advantage over overt only
	No difference in detection accuracy	This advantage is not due to a speed–accuracy trade-off
Spatial distribution of fixations	More fixations between stimuli in E2 than in E1	Untethered covert and overt attention resulted in a different fixation strategy

**Table 2:** Indices of implicit processing provided by a comparison of catch trials with change blindness (CB) trials

Measure	Result	Interpretation
Main evidence for implicit processing		
Change detection time	Catch trials are slower than CB trials	Unlike pseudo targets on catch trials, real targets attract attention even though the change is not detected.
Fixations on target	More fixations on target on CB than catch trials	
First fixation on target*	Shorter (E1 only) and earlier on CB than on catch trials	
Duration of fixations on target	Shorter fixations on CB than catch trials	Undetected change continues to attract attention. However, the real target receives more shallow processing compared to other items.
Time on target immediately before trial end	Less mouse/gaze time on target for CB than catch trials	
Gaze-to-target distance	Shorter on catch than CB trials	No items close to the target attract attention and/or observers on catch trials engage in serial analysis of items.
Saccade amplitude†	Higher on CB compared to catch trials (E1 only)	More overt attention on target when target is first processed via covert attention.
Pupil size at the end of the trial†	Smaller on catch than on CB trials (E1 only)	The additional engagement of covert attention corresponds to increases in pupil size.

*Note*. E1 = Experiment 1; * Significant differences between experiments; ^†^ Some effects are present in only one of the experiments, but there are no significant between-experiment differences

### Decoupling attention: Change detection efficiency

Observers found the target faster in Experiment 1 (*M* = 8.00 s [7.41, 8.59]) than in Experiment 2 (*M* = 10.43 s [8.10, 12.75]), *t*(21.6) = −2.12, *p* = .046, on trials when it was found before the liking procedure started (square brackets denote 95% confidence intervals; see Table S1 in the Supplement for descriptive statistics). Search was similarly faster in Experiment 1 than in Experiment 2 when the target was reported after the liking procedure started (*M* = 8.01 [6.74, 9.50] vs. *M* = 10.40 [9.42, 11.53]), *t*(50.8) = −2.68, *p* = .010. Accuracy was calculated as the summed share of trials when the target was identified either during the main part of the trial or during the liking procedure and was similar in both experiments (55% in Experiment 1, 56% in Experiment 2). The target was selected more often during the liking procedure in Experiment 2 than in Experiment 1. The number of false alarms during either the main part of the trial or the liking procedure was low in both experiments, (3% in Experiment 1, 2% in Experiment 2). Some participants in Experiment 2 (*N =* 6) selected the target during the liking procedure instead of indicating that they found a target by pressing the response key on more than 90% of trials. The rest of their trial outcomes were distributed similarly to other observers, so these observers were excluded only from further analyses of “target found” trials. The probability of change blindness (CB), that is, when observers positioned the cursor or looked at the target but did not indicate that they found the change and thereby starting the liking procedure, was 18% in Experiment 1 and 19% in Experiment 2.

### Decoupling attention: Fixation dispersion

**Fig. 2:**
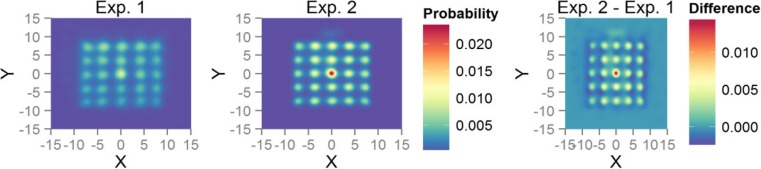
Spatial map of fixation densities. Warmer colors show higher probability of fixation at that particular point. Difference plot shows difference between Experiment 2 and Experiment. 1, with warmer colors indicating that the probability of fixation at each respective point is higher in Experiment 2. Plots show that in Experiment 1, fixations are more widely spread and there are more fixations in between stimuli than in Experiment 2. (Color figure online)

Trials where no fixations at all were detected were excluded from fixation analyses. To estimate the difference in the observed spatial distribution of fixations (see Fig. 2), we computed the frequencies of fixations within a 15 × 15 degrees of visual angle square zone at display center split into 100 × 100 bins. Syrjala’s (1996) test (implemented in the *ecespa* library in R; de la Cruz Rot, 2008), which provides permutation-based estimates of the difference between two spatial point distributions (based on Cramer–von-Misses and Kolmogorov–Smirnov tests), revealed that the fixation distributions in Experiment 1 differed significantly from Experiment 2 (psi = 33, *p* < .001 for Cramer–von-Misses test; psi = 0, *p* < .001, for Kolmogorov–Smirnov test). A comparison of the average share of fixations on stimuli between observers showed that in Experiment 1, observers were less likely to fixate on stimuli than in Experiment 2 (*M* = 0.46 [0.41, 0.50] vs. *M* = 0.79 [0.77, 0.82], *t*(45.5) = −11.85, *p* < .001).

### Implicit processing: Trial duration and time spent on target

Both trial duration and time spent on target differed between genuine CB trials and catch trials. In Experiment 1, CB trials were on average 10.5 [8.51, 12.48] seconds shorter, *t*(31) = 10.76, *p* < .001, and observers spent 91 [52, 131] ms less on the target, *t*(31) = 4.75, *p* < .001. Similarly, in Experiment 2, CB trials were again shorter, *M* = −7.57 [−8.95, −6.20], *t*(23) = 11.41, *p* < .001, and observers spent less time on the target, *M* = −91.46 [−130.75, −52.16], *t*(31) = 4.75, *p* < .001. We used linear mixed-effects regression (LMER) with random intercepts and slopes for each subject to test for the relationship between trial outcome and time spent on target. The R library lme4 (Bates, Mächler, Bolker, & Walker, 2015) does not provide *p* values for LMER, but values of *t* above 2 can be used as a guide for significance of the results (comparable to alpha = .05 for *p* values). Importantly, LMER showed that the difference in time spent on the target between CB trials and catch trials remained significant when trial duration was controlled for, both in Experiment 1, *B* = 0.11 (0.03), *t* = 3.33, and in Experiment 2, *B* = 0.15 (0.04), *t* = 3.34, which strongly suggests implicit processing of unnoticed changes.

### Implicit processing: Fixations on target

**Fig. 3:**
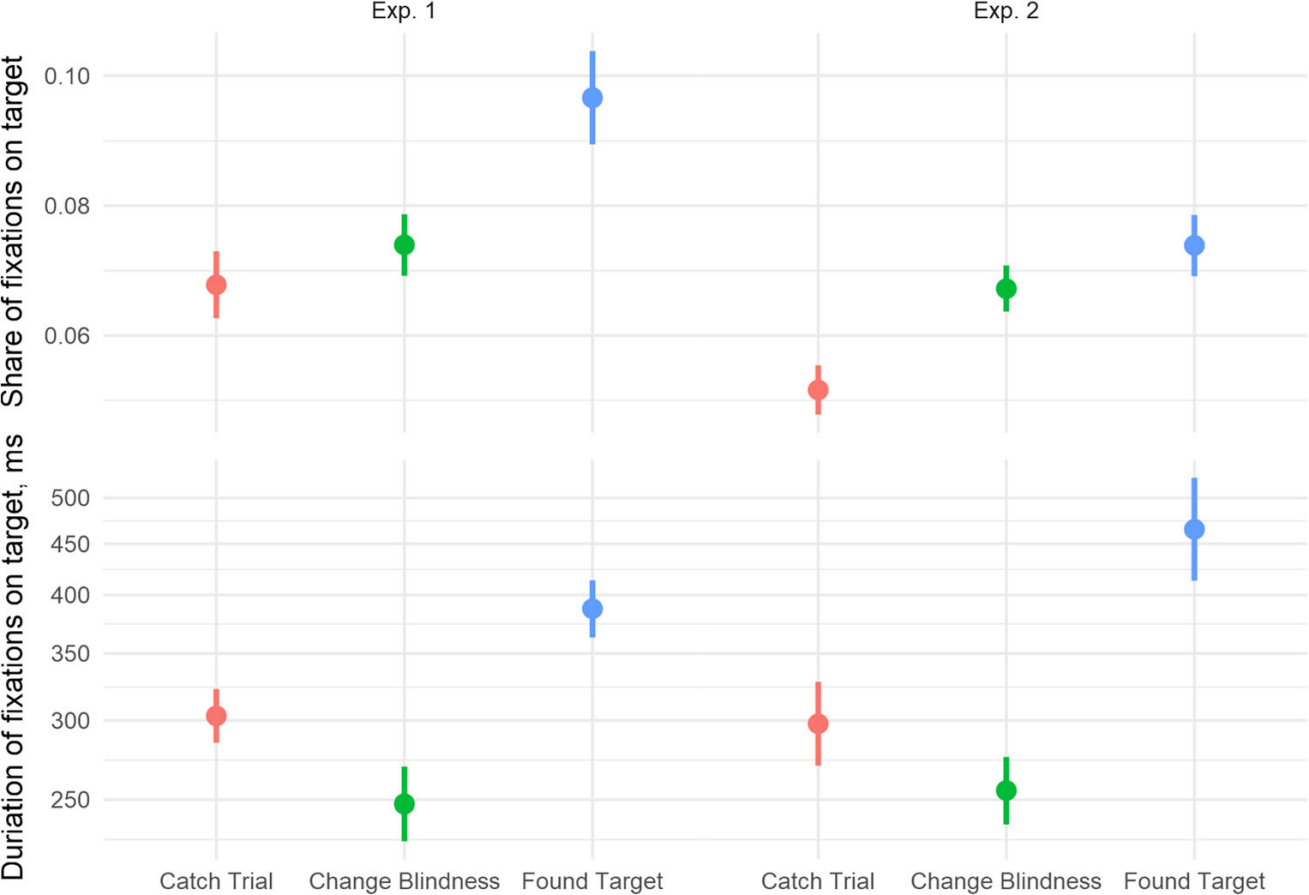
Fixation probabilities and durations. Error bars show 95% confidence intervals. Participants in both experiments were more likely to fixate the target on FT trials, and those fixations tended to be longer. On CB trials, fixations were more frequent but of shorter duration than catch trials, but this was only significant in Experiment 1

We analyzed the raw data with LMER without aggregation by trial and thus had no need to exclude any trials from the fixation analyses, except for the share of fixations on target (variability between participants was accounted for by including random effects for participants and appropriate random slope effects). For the share of fixations on target, trials were included with share of fixations on target equal to zero. In Experiment 1, the average probability of fixations on the target (the proportion of the total fixations within a trial that were on the target) was lower for catch than for CB trials, *t*(30.0) = −2.37, *p* = .025, which was in turn lower than for FT trials, *t*(30.0) = −5.56, *p* < .001 (see Fig. 3). The mean number of fixations on the target per trial was 2.28 [2.15, 2.40] for catch trials, 1.06 [1.00, 1.12] for CB trials, and 1.53 [1.49, 1.57] for FT trials. The duration of those fixations was, on the other hand, lower for CB than for catch trials, *B* = −0.18 (0.03), *t* = −6.43, and higher for FT than catch trials, *B* = 0.27 (0.02), *t* = 11.13 (trial duration controlled for).

In Experiment 2, again, the average probability of fixation on the target was lower for catch than for CB trials, *t*(23.0) = −5.49, *p* < .001, which was, in turn, numerically but not significantly lower than for FT trials, *t*(17.0) = −1.37, *p* = .188. The mean number of fixations on the target per trial was 1.79 [1.72, 1.86] for catch trials, 1.44 [1.37, 1.51] for CB trials, and 1.40 [1.36, 1.44] for FT trials. As in Experiment 1, the duration of those fixations was lower for CB than catch trials, *B* = −0.17 (0.04), *t* = −4.01, and higher for FT than catch trials, *B* = 0.41 (0.04), *t* = 10.17.

Between-experiment comparisons showed that for the share of fixations on target, both the effect of experiment, *F*(1, 47) = 4.86, *p* = .032, η^2^_G_ = 0.07, and its interaction with trial result were significant, *F*(2, 94) = 5.76, *p* = .006, η^2^_G_ = 0.03. The average share of fixations on target was higher in Experiment 1 than Experiment 2 on FT trials, *t*(38.8) = 3.30, *p* = .002, but not on CB trials, *t*(42.9) = 0.95, *p* = .347. For fixation duration, there was a significant interaction between trial result and experiment, showing that the duration of fixations on FT trials compared to duration on catch trials increased more in Experiment 2 than in Experiment 1, *B* = 0.20 (0.04), *t* = 4.87.

**Fig. 4:**
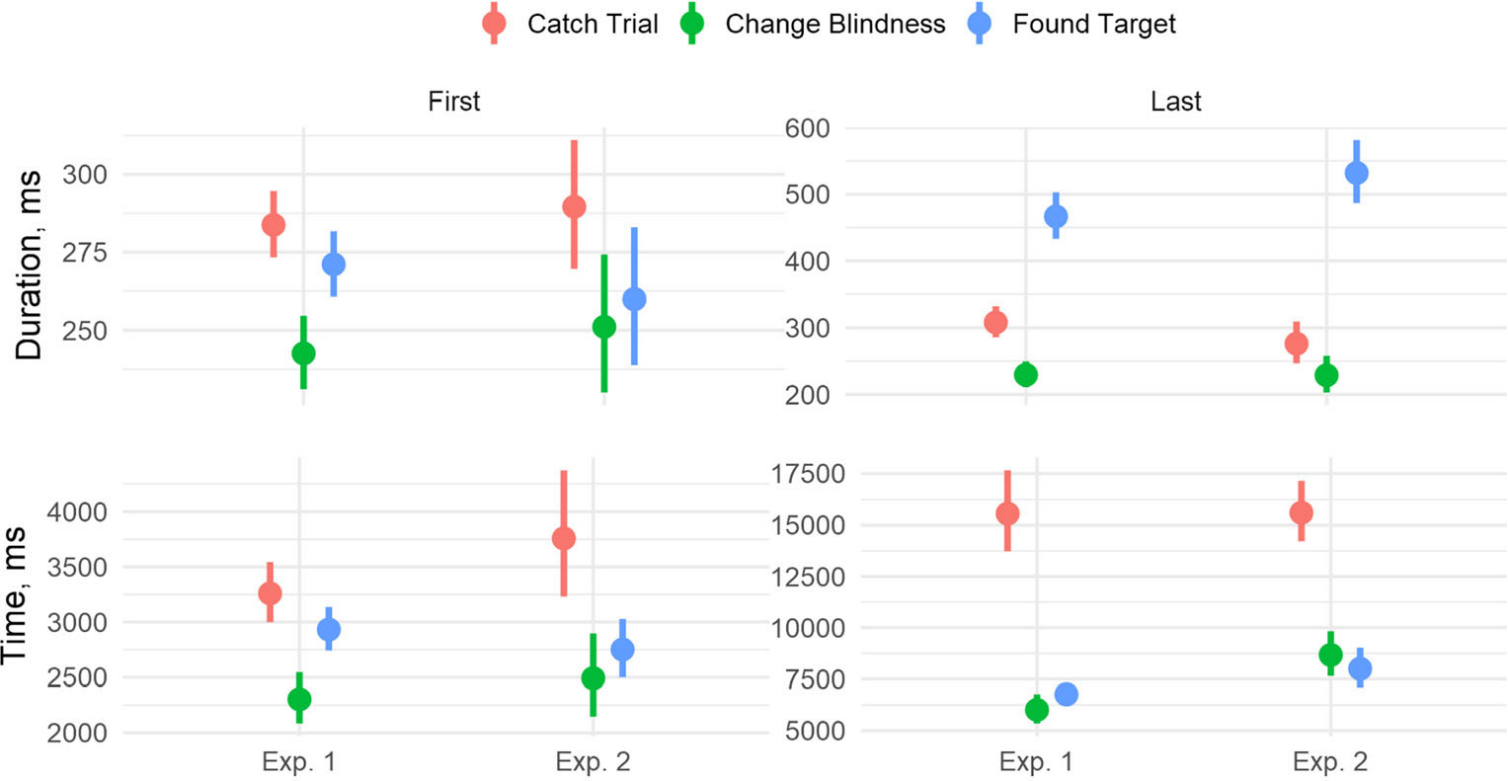
Comparison of first and last target fixations. Data were analyzed log transformed, the plots show untransformed values for clarity. Bars show 95% confidence intervals. (Color figure online)

We also compared first and last target fixations by trial outcome. Speculatively, implicit processing of targets might occur on the first fixation on it (and then if a change is missed the target may be ignored afterwards) or on the last fixation (that corresponds to explicit analyses of the target when the change is found). Interestingly, Fig. 4 shows that first fixations on the target in Experiment 1 on CB trials were shorter, *B* = 0.10 (0.04), *t* = 2.63, and occurred earlier, *B* = 0.28 (0.08), *t* = 3.50, than first fixations on catch trials. The same goes for the last fixation, *B* = 0.14 (0.05), *t* = 2.85, for duration, and *B* = 0.90 (0.09), *t* = 9.62, for time. Compared to FT trials, however, the first fixations were earlier, *B* = 0.19 (0.07), *t* = 2.57, but not shorter, *B* = 0.05 (0.04), *t* = 1.35, on CB trials, and the last fixations were shorter, *B* = 0.68 (0.05), *t* = 13.23, but not earlier, *B* = 0.06 (0.06), *t* = 1.11. Note that the effect of fixation time relative to trial start is controlled for in the duration analyses.

In Experiment 2, the first fixations on CB trials occurred earlier, *B* = 0.33 [0.10], *t* = 3.47, and were nonsignificantly shorter, *B* = 0.11 (0.06), *t* = 1.68, than the first fixations on catch trials. The last fixations, however, were both shorter and occurred earlier, *B* = 0.22 (0.08), *t* = 2.65, for duration, and *B* = 0.57 (0.06), *t* = 9.24, for time. Compared to FT trials, the first fixations were neither earlier, *B* = 0.03 (0.09), *t* = 0.36, nor shorter, *B* = 0.02 (0.06), *t* = 0.33, on CB trials, but the last fixation was shorter, *B* = 0.81 (0.08), *t* = 9.69, but not earlier, *B* = −0.12 (0.07), *t* = −1.85.

Comparisons between experiments showed that the final, *B* = 0.29 (0.09), *t* = 3.32, but not first fixations, *B* = 0.11 (0.09), *t* = 1.21, occurred earlier in Experiment 1 than in Experiment 2. However, the differences in fixation duration between experiments were not significant (*t* < 0.6).

**Fig. 5:**
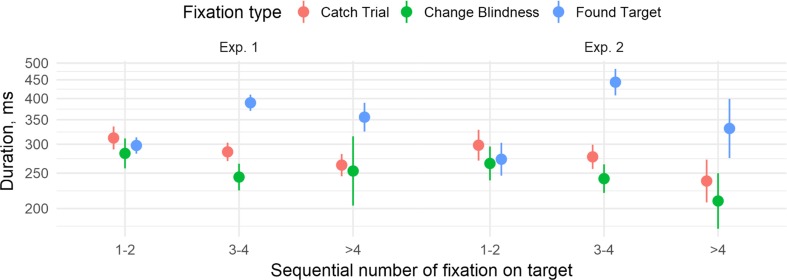
Estimated marginal means for fixation duration with repeated fixations on target controlling for fixation start time. Bars show 95% confidence intervals. For catch trials and CB trials, the fixation duration decreased with repetitions, but on found target trials the effect was reversed. (Color figure online)

Fixation durations decreased when observers repeatedly fixated on the target during a trial, even when controlling for fixation start time. In Experiment 1, each subsequent target fixation decreased in duration, *B* = −0.09 (0.03), *t* = −2.99. Importantly, trial outcome interacted with fixation number: The change in duration with repetition was significantly more positive for FT, *B* = 0.17 (0.04), *t* = 4.81, than for CB trials, but the difference between CB and catch trials was not significant, *B* = 0.06 (0.03), *t* = 1.85. As Fig. 5 shows, fixations on FT trials become longer when repeated, in contrast to fixations on catch and CB trials. In Experiment 2, the overall fixation duration decrease was not significant, *B* = 0.04 (0.03), *t* = 1.52. The interaction analyses indicated that repeated fixations on FT again become longer, *B* = 0.27 (0.05), *t* = 5.07, but the difference between the rate of duration decrease between CB trials and catch trials was not significant, *B* = −0.01 (0.03), *t* = 0.34. The analysis of the two experiments together yielded the same results with a nonsignificant difference between them (all *t*s < 1.2).

### Implicit processing: Saccade amplitude

**Fig. 6:**
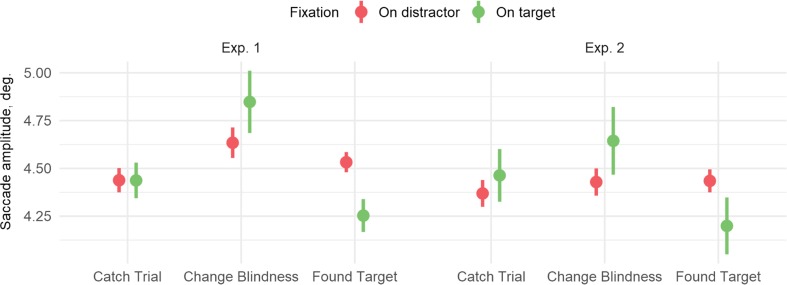
Saccade amplitudes as a function of trial outcome. Error bars show 95% confidence intervals. Saccade amplitudes preceding fixations on targets tend to be higher for change blindness trials than for catch trials or “found target” trials (the former is significant in Experiment 1 but not Experiment 2). (Color figure online)

In both experiments, the pattern of saccade amplitudes shows that CB trials differ from the other two trial types (see Fig. 6). In Experiment 1, fixations on “targets” for catch trials were preceded by lower amplitude saccades when compared to CB trials, *B* = 0.19 (0.09), *t* = 2.14, and higher amplitudes compared to FT trials, *B* = −0.34 (0.07), *t* = −5.10 (the difference in the time relative to the beginning of the trial is accounted for). In Experiment 2, the pattern was the same, but only the difference between catch and FT trials was significant, *B* = −0.35 (0.10), *t* = −3.49, but not the difference between catch and CB trials, *B* = 0.13 (0.12), *t* = 1.09. There was also a nonsignificant amplitude difference for saccades to stimuli other than targets (all *t*s < 1.3). Moreover, when both of those saccade types were analyzed together, there was a significant interaction for trial type (CB vs. catch vs. FT) and fixation type (fixation on target vs. fixation on other stimuli). For Experiment 1, the interaction term was significant for CB versus catch trials, *B* = 0.24 (0.10), *t* = 2.53, and for catch trials versus FT trials, *B* = −0.20 (0.06), *t* = −3.18. For Experiment 2, only the latter term was significant, *B* = −0.27 (0.07), *t* = −3.94; *B* = 0.10 (0.08), *t* = 1.22, for catch versus FT. When the two experiments were analyzed together, however, the overall difference between them was not significant (all *t*s < 1.7).

### Implicit processing: Gaze-to-target distances

**Fig. 7:**
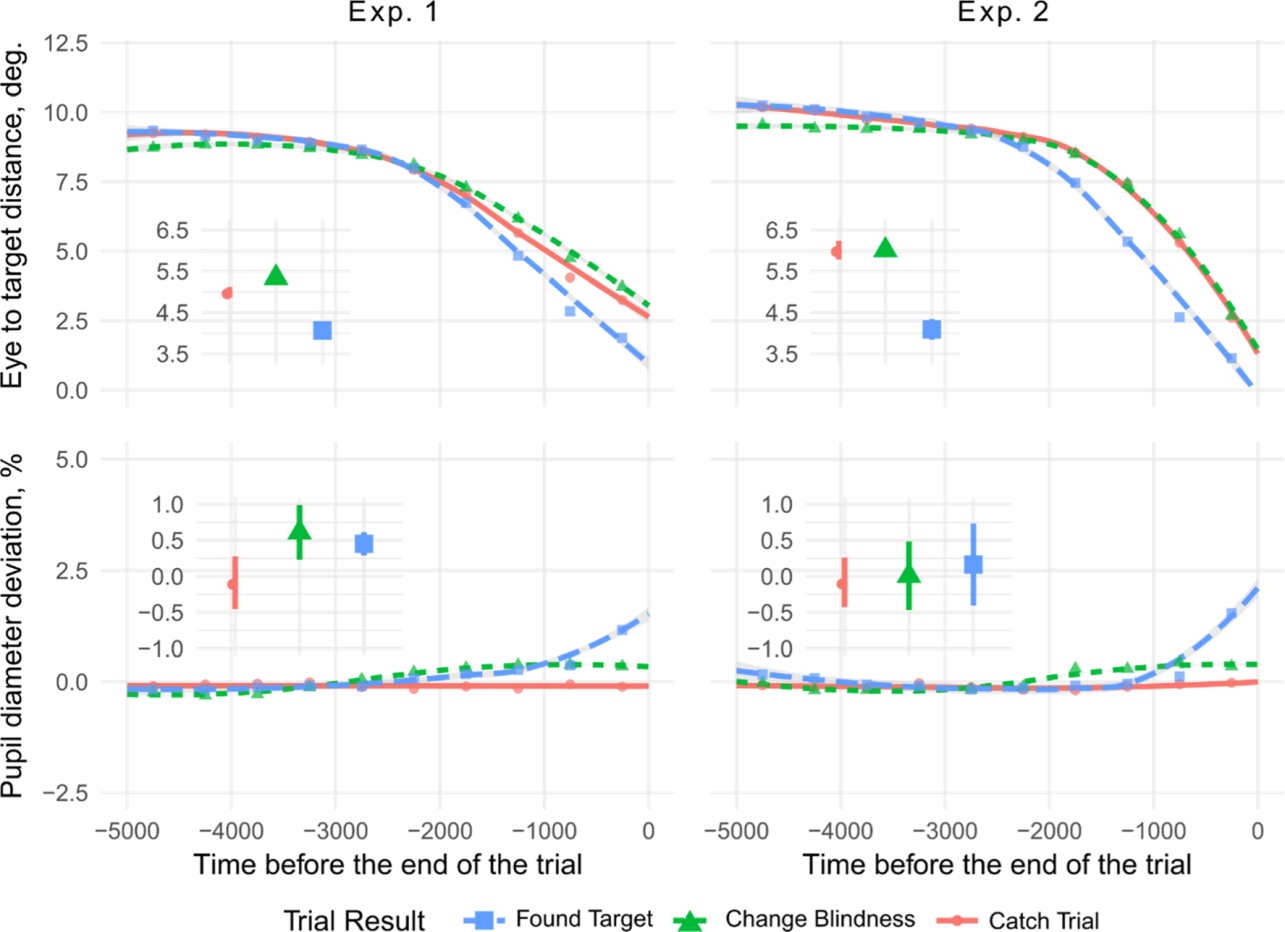
Pupil diameter and distances to gaze positions as a function of time before trial end. In the line plots, dots show mean values, lines show nonlinear smoothed trends. Subplots show mean values within the last 2,000 ms, with 95% within-subject confidence intervals as lines. Panels show that the distance between the target and gaze positions decreased toward the end of trials. Differences between trials are readily observed starting approximately 2 seconds before the end of the trial, and therefore we used this period as a limit for our further analyses. (Color figure online)

In addition to the last fixations, we analyzed the distance between gaze location and target during the last seconds preceding the end of each trial (see Fig. 7). LMER with random effects for observer and outcome by observer showed that in Experiment 1, gaze position was farther from the target on change blindness than on catch trials, *B* = 0.35 (0.10), *t* = 3.59, and if the target was found, gaze-to-target distance was smaller than on catch trials, *B* = −0.93 (0.12), *t* = −8.04. In Experiment 2, however, average distance to gaze position did not differ between CB and catch trials, *B* = −0.01 (0.11), *t* = −0.09. This can be expected as both catch and change blindness trials in Experiment 2 ended right after observers looked at the target and then shifted their gaze. On FT trials, average distance to gaze position was smaller than on catch trials, *B* = −1.91 (0.18), *t* = −10.59.

Direct comparisons between experiments showed significant interactions between experiment and trial type, *B* = −0.36 (0.15), *t* = 2.40, for CB against catch trials interaction with experiment, and *B* = −0.95 (0.21), *t* = 4.62, for “found target” against catch trials interaction with experiment. Pairwise comparisons showed that the average distance to gaze position was higher in Experiment 2, both on catch, *B* = 1.02 (0.36), *t* = 2.82, and CB trials *B* = 0.65 (0.31), *t* = 2.12; but on FT trials, the difference was not significant, *B* = 0.06 (0.37), *t* = 0.17. Again, these differences between experiments are expected because catch trials and CB trials end when the cursor is on the target in Experiment 1 and when gaze is on the target in Experiment 2.

### Implicit processing: Pupil diameter

Figure 7 also shows differences in pupil size as a function of trial outcome. We computed average pupil size for each observer on each trial during the period from 2 to 5 seconds before the end of the trial that served as a baseline. The deviation in pupil diameter was then computed with PD_dev_ = (PD − PD_base_)/PD_base_PD_dev_ = (PD − PD_base_)/PD_base_, where PDPD is pupil diameter and PD_base_ is the baseline pupil diameter.

When observers found the target in Experiment 1, there was an increase in pupil diameter before the end of the trial, *B* = 0.54 (0.20), *t* = 2.69. Moreover, the pupil diameter was larger on CB than catch trials, *B* = 0.69 (0.35), *t* = 2.00, again indicating notable differences between CB trials where the change is not noticed and trials without a change, revealing implicit processing of changes. In Experiment 2, there was again an increase in pupil diameter before trial end on FT trials, *B* = 0.57 (0.19), *t* = 3.02, but the difference between CB and catch trials was not significant, *B* = 0.11 (0.33), *t* = 0.34. The interaction between experiment and trial result, was not significant (*t* < 1.25).

### Implicit processing: Liking and choice bias

Using liking procedures with two question types (liked most vs. liked least) allowed us to disentangle choice biases from changes in evaluation (Chetverikov & Kristjánsson, 2015). Both on CB and on catch trials, targets were evaluated more positively than were distractors (i.e., selected earlier when asked “Which one do you prefer the most” and later when asked “Which one do you prefer the least”), but the difference was significant only in Experiment 2 (*M* = 0.07 [0.02, 0.12], *t*(23) = 3.10, *p* = .005). Analysis of choice biases toward targets (i.e., whether a target is selected earlier than distractors independently of the question asked) did not show any differences in either experiment.

A comparison of CB and catch trials did not reveal any difference in liking or choice biases. In Experiment 1, the liking ratings for targets on CB and catch trials were similar, *B* = −0.00 (0.03), *t* = −0.04, while biases were numerically more pronounced on CB trials, *B* = −0.02 (0.04), *t* = −0.41. In Experiment 2, the liking ratings for targets on catch trials were numerically more positive, *B* = 0.02 (0.04), *t* = 0.56, while biases were numerically more pronounced, *B* = 0.03 (0.05), *t* = 0.73, than on CB trials.

## Discussion

We compared change blindness trials with catch trials and trials where the target was found in a change-blindness paradigm with a mouse-contingent (Experiment 1) and a gaze-contingent display (Experiment 2). We aimed to assess implicit target processing prior to the first “attentive blank stare” and to analyze effects of partially limiting the dissociation between overt and covert attention on change blindness.

We highlight the following results.

1. Our findings are the first to show that partial decoupling of covert and overt attention leads to faster change detection, and our eye-gaze measurements provide suggestions about how this may occur. Gaze can be dispersed more diversely when covert attention is separated from the locus of gaze, as Fig. 2 shows, and our results also suggest that this aids change detection.
2. We present highly detailed analyses of eye-gaze patterns, which yield the most compelling eye-monitoring evidence for implicit change detection so far available in the literature. For example, we show that unlike pseudo targets (on catch trials), real targets attract attention, even if the change is not detected and how pupil size changes immediately before the first “attentive blank stare.”
3. Our results show that unnoticed targets are processed but also that the processing is likely shallower than if the changing target is consciously detected. The first fixations on target on change blindness trials are particularly revealing, as they occur earlier and are shorter than on both catch trials and found target trials. In what follows, we go over these points in more detail.

### Decoupling overt and covert attention

The comparisons between mouse-contingent (Experiment 1) and gaze-contingent displays (Experiment 2) indicate that participants search for a change differently when they can deploy covert attention separately from gaze position. By untethering the viewing area from the focus of gaze position, participants were free to position the center of the Gaussian window in a way that allowed them to deploy covert attention where they saw fit. In support of the effectiveness of this manipulation for attention, we found that observers in Experiment 2 fixated on stimuli more often, while in Experiment 1 the distribution of fixations dispersed more widely, including into areas in between the stimuli. In other words, with mouse-contingent displays, observers were more likely to fixate between the stimuli, presumably because they could use covert attention to analyze the actual stimuli. The results also confirmed our prediction that the gaze-contingent paradigm should increase change detection times because the mouse-contingent display allowed independent allocation of overt and covert attention (Belopolsky & Theeuwes, 2009; Hunt & Kingstone, 2003; Kristjánsson, 2011; Posner, 1980; Walter et al., 2012). Tas, Luck, and Hollingworth (2016) showed that covert shifts of attention in a color change detection task yielded no performance costs. Furthermore, Jonikaitis and Deubel (2011) demonstrated that observers could allocate hand and eye movements in visual search to separate locations resulting in synergy when the two were coordinated. Accordingly, while overt attention was deployed at gaze position in both experiments, covert attention could be deployed independently of the gaze position in Experiment 1, with the mouse-contingent display, facilitating change detection.

There were no differences in change blindness rates between the two displays, however. That is, even though observers spent more time looking for changes with gaze-contingent than mouse-contingent displays, they were as likely to miss the change with both types. In our study, a trial was categorized as a change blindness trial when the cursor (Experiment 1) or gaze (Experiment 2) remained within 2.5° or less from the target center for long enough for both target versions to be presented for at least one frame followed by a mouse movement or gaze leaving the target area. That is, we classified trials as “change blindness” when “attentive blank stares” occurred (as indicated by gaze or mouse position^3^). Our predictions regarding the effect of untethered overt and covert attention on the probability of “attentive blank stares” were therefore not supported.

It is possible that with the mouse-contingent display, observers had increased attentional load because they had to control the viewable area independent of their gaze location. However, we believe that this is unlikely because mouse-based operations are ubiquitous in modern environments, and, hence, controlling mouse position is a highly automatized skill. Moreover, attentional load is known to decrease performance on perceptual awareness (Lavie, Beck, & Konstantintou, 2014), so we would expect a decrease in change detection performance in the mouse contingent condition if this were the case. Our results show an improvement in performance in the mouse contingent display, so differences in attentional load are unlikely as a cause.

Caplovitz et al. (2008) argued that attentive blank stares might result from an uneven spread of attention between objects and features at fixation, stating that “observers orient their gaze and spatial attention towards the location of the change, but focus a higher-order object-based form of attention on features or objects within that location that are not actually changing” (Caplovitz et al., 2008, p. 885). While it might be true in natural scenes, when a hierarchy of features defines objects, in our experiment, features (shape, color, figure) contributed equally to the object identity, and one could not be preferred over the other. Given the lack of a convincing explanation, we believe that “attentive blank stares” warrant further investigation.

### Implicit and explicit target processing

We suggest that differences between found-target and change blindness trials represent explicit target processing that reaches participant’s awareness. On trials where the target was found, observers fixated on the target more often, the fixations were longer, and the saccades to the target were initiated from shorter distances. Moreover, the average gaze-to-target distance at the end of a trial was smaller on trials with successful change detection in comparison to change blindness trials and trials with no changing item. This is in agreement with other studies and indicates that spatial proximity to gaze is a crucial factor for change detection in flicker paradigms (Henderson & Hollingworth, 1999; Hollingworth et al., 2001; Vachon et al., 2012). In addition, successful target detection correlates with pupil size, similar to earlier results (Privitera, Renninger, Carney, Klein, & Aguilar, 2010; Vachon et al., 2012). In sum, we found clear evidence of target detection in the eye-movement data that reflect arousal and decision-making in the presence of the target.

More importantly, several indices in both experiments reveal implicit processing of unreported changes. This question of whether change detection can occur implicitly has been discussed extensively in the literature (Fernandez-Duque & Thornton, 2000, 2003; Mitroff & Simons, 2002; Tseng et al., 2010). We propose that differences between change blindness and catch trials in our paradigm represent implicit processing since the change fails to sufficiently reach awareness for report. While the pattern of results related to these indices is undeniably complex (see Table 2 for a summary), we propose a simple interpretation based on three main findings.

First, we found that observers fixate targets earlier on change blindness trials than foil “targets” on catch trials. Observers attend early to the changing target’s position even though they do not notice the change and continue attending that position throughout the trial, without noticing the change. Note, however, that these fixations are not “attentive blank stares”—they do not continue throughout the blank interval (such fixations would end the trial). These results are one of the key findings here, indicating that the change attracts attention independently of whether it is found or not, but when the change is missed, only one version of the changing target is processed on each fixation.

Second, when observers miss changes, their saccades to targets seem to have larger amplitudes than when the change is found. Moreover, the average distance between eye and target during the last seconds of trials is larger when observers do not detect a change. The increase in saccade amplitude when change detection fails is similar to the findings of Henderson and colleagues (Henderson & Hollingworth, 1999, 2003). Larger saccade amplitudes indicate that saccades are initiated when the target is more peripheral in the visual field.

Third, crucially, fixation durations on targets are shorter on change blindness than on found-target trials and may therefore not allow participants to catch both versions of the changing target on each fixation. The difference in durations is already evident for the first fixation on the target and the durations continue to decrease over the course of a trial. This indicates that target processing on change blindness trials is shallow and becomes shallower as the trial proceeds.

In sum, we suggest the following scenario: Unnoticed changes attract attention early, but this is likely to happen when the target is in the visual periphery (hence the long saccades), and when the target is subsequently fixated, its processing is shallow. This pattern repeats, leading to further decreases in target fixation duration, which further decreases target processing, and the chance that both versions of the target will be processed during one fixation. According to Mitroff, Simons, and Franconeri (2002), “successful change detection requires observers to both form a representation and compare that representation [of the prechange information to the postchange information]” (p. 799). Our findings show that while implicit change detection does occur (otherwise, the change would not attract attention), the processing of the change is too shallow for explicit change detection, and observers do not have representations of both the prechange and postchange information.

Additional support for processing of unnoticed targets was found in Experiment 1, where change blindness was accompanied by increases in pupil size. It is well-known that pupil dilation depends on a variety of cognitive factors, including mental effort and task load (Alnæs et al., 2014). Changes in pupil size can also indicate a switch of attention focus (Einhäuser, Stout, Koch, & Carter, 2008) or uncertainty in decision-making and subconscious decisions (Privitera et al., 2010), which might take place during change blindness tasks. So in line with Vachon et al. (2012), we can assume that despite a failure to find the change, it was nevertheless processed to some extent. This nonexplicit “shallow” processing on change blindness trials may indicate an orienting period in target search (Karpov, Luria, & Yarbus 1968) or ambient attention (Unema, Pannasch, Joos, & Velichkovsky, 2005) and is certainly different from the focal attentive processing with long fixations and a short succession of saccades.

Notably, the results that suggest implicit target processing cannot be explained away by the differences in strategies employed by observers on different trials. For example, observers might engage in systematic analysis on some trials (shorter saccades, longer dwell times, and, later first fixations) while other times scanning rapidly (longer saccades, shorter dwell times). One could argue that the former strategy might have an increased likelihood of finding the target (although we are not aware of any evidence clearly supporting that). Then, CB trials would have shorter than average fixations, and found trials would have longer than average fixations. Catch trials serve as an average in this case since they would be a mix of both strategies. The observed differences in average fixation durations are consistent with this explanation. However, the rest of the data show a different pattern. Consider the data on first fixations: The onset time and the duration of first fixations on CB trials and on “found target” trials are lower than on catch trials. If the proposed strategic explanation were correct, we would expect to see late and long first fixations on “found target” trials, which was not the case. Moreover, a difference in average fixation durations is much more readily explained by the explicit processing of target when the change was detected. This is evident from the data on the last fixations showing the effect of postdetection processing on fixation durations.

Although the main effects of performance (detection and fixation dispersion) show an impact of untethering covert and overt attention, the role of covert attention on implicit processing is less clear. Hints of interactions between experiment and trial result suggest that being able to dissociate covert attention from gaze position might partially facilitate implicit detection on change blindness trials, however most of these interactions were not significant when compared across experiments. Additionally, the fact that catch trials and change blindness trials ended when observers gaze or the cursor landed on the target and afterwards moved it away can explain the differences in eye-to-target distance. However, Johnson, Mulder, Sijbinga, and Hulsebos (2012) have shown that using a mouse does not affect the viewing patterns of observers, suggesting that the main effects of covert attention on change detection time and fixation dispersion are real.

The present results also contribute to the growing body of literature on implicit change detection by using conscious and precise change localization instead of guessing. It is useful to compare our study to previous studies of change detection that also utilized mouse clicks or motor actions. For example, Mitroff and Simons (2002) and Clark, Fleck, and Mitroff (2011) asked observers to use mouse clicks to indicate the possible change location after each presentation cycle. In contrast, Tseng et al. (2010) presented changed and nonchanged versions of the image once, and then asked observers to point out the changed location. The procedure we used does not involve constant interruptions, produced by repeated estimation of the change location as in the former example, and does not require correction for guessing rates as in the latter.

Note that “attentive blank stares” can occur not only in change blindness studies but also in inattentional blindness (Beanland & Pammer, 2010; Pappas, Fishel, Moss, Hicks, & Leech, 2005; Richards, Hannon, & Vitkovitch, 2012; Simons & Chabris, 1999). In such studies, there might be different factors at play, such as observers’ expectations or an incongruency between target and task set. Some of these factors, such as expectations, especially similarity between target and nontargets or expectations based on other items in the display, may also affect “blank stare” rates in change blindness. Further studies could provide additional insights by explicitly comparing the two paradigms using similar displays.

Finally, we did not find any evidence for affective changes for targets on change blindness trials. While for visual search observers dislike targets when they look at them but do not recognize them as targets (Chetverikov et al., 2015), here, observers showed increased preferences for both targets and distractors. One difference between the two paradigms is that the stimulus set in this study was smaller and the stimuli were repeated during the experiment. However, Chetverikov and Kristjánsson (2015) tested preferences for repeating stimuli and found devaluation related to the conflict in visual search. Note that the positive evaluation effect reached significance only in Experiment 2, and this was also associated with longer times spent on the target before the liking procedure started. This positive evaluation may therefore be similar to “mere exposure” effects (Bornstein, 1989; Zajonc, 1980, 2001). Speculatively, this might indicate that comparisons between two versions of the changing stimuli do not occur, supporting ideas put forward by Beck and Levin (2003) and Mitroff et al. (2004). That is, while representations of target stimuli are retained (causing more positive evaluation), the comparison that might lead to a conflict and negative affect does not occur. According to the affective feedback for perceptual predictions model (Chetverikov & Kristjánsson, 2016), observers do not predict one version of the stimuli based on another. One way to test this idea further in future studies would be to compare preferences for targets with different time intervals between the two versions of the changing target.

## Conclusions

The pattern of results emerging from our analysis of eye movements and behavioral data supports the idea of implicit target detection on change blindness trials. Crucially, our results also cast light on why this may occur by providing a highly detailed picture of eye movements during trials. Even when observers fail to identify the target, their spatial attention (and gaze) is nevertheless pulled toward the change position, and the target is processed to some extent. The dissociation of covert and overt attention with gaze and mouse contingent displays affects the efficiency of change detection and the spatial distribution of fixations, but does not change the probability of “attentive blank stares” or the observed patterns of the indices of implicit processing. Although we were unable to establish a connection between the deployment of covert attention and implicit processing, we believe the new methodology that we use has the potential for more precise investigations in future studies. Finally, our results suggest that the absence of change awareness in the presence of indices of implicit processing in behavioral and eye-movement data is not related to a dissociation of overt and covert attention.

### Author note

The studies reported in this article were supported by Russian Foundation for Basic Research (#15-06-09321А) and Icelandic Research Fund (IRF #152427)

## Electronic supplementary material

ESM 2 (DOCX 3827 kb)

## Footnotes

1 Another kind of gaze-contingent display is used in transsaccadic change blindness studies, where some element of the display is changed during a saccade (see reviews of transsaccadic memory in Higgins & Rayner, 2015). Such procedure is quite different from the one we use, as the change itself is contingent on eye movements.

2 Except for catch trials, where no items changed.

3 Note that analyses of fixations did not reveal any difference between fixation durations within “attentive blank stare” periods between the experiments (average fixation duration on target on change blindness trials within this period was 319 ms in Experiment 1 and 336 ms in Experiment 2; no significant differences were found in analyses of fixation duration, amount, or distance to targets). This shows that even though mouse location was used as a criterion for ending the trial in Experiment 2, mouse location was linked to gaze location, and the “attentive blank stares” are comparable between the experiments.
