# Supplementary material for: Implicit processing during change blindness revealed with mouse-contingent and gaze-contingent displays
Source: Atten Percept Psychophys. 2018 Jan 23;80(4):844–59. doi: 10.3758/s13414-017-1468-5 (PMC5948240; doi:10.3758/s13414-017-1468-5)
Supplement: Supplementary file 2 — (DOCX 3827 kb) [file 13414_2017_1468_MOESM2_ESM.docx]

# Supplement

Table S1. Descriptive statistics for each possible trial outcome. N - average number of trials per observers. Trial Duration - time before observer indicated that the target is found or before the liking procedure is started. Time on Target - time mouse cursor or gaze was at the target position before leaving it and thus starting the liking procedure. Liking - liking rating (higher means more positive). Bias - target item rank in liking procedure regardless of scale direction. SDs are shown in parentheses

| Experiment | Trial Outcome | Share | Trial Duration, sec | Time on Target, ms | Liking | Bias |
| --- | --- | --- | --- | --- | --- | --- |
| Exp. 1 | Found Target | 0.52 (0.08) | 8.0 (1.6) |  |  |  |
|  | False Alarm | 0.02 (0.04) | 20.0 (23.2) |  |  |  |
|  | Selected Later | 0.04 (0.03) | 8.0 (3.8) | 611 (353) |  |  |
|  | Selected Later, Incorrectly | 0.00 (0.01) | 8.1 (5.4) | 539 (250) |  |  |
|  | Liking Procedure (CB trial) | 0.18 (0.07) | 7.1 (1.8) | 571 (133) | 0.03 (0.16) | 0.04 (0.18) |
|  | Liking Procedure on Catch Trial | 0.24 (0.02) | 17.6 (5.0) | 663 (130) | 0.01 (0.11) | 0.02 (0.16) |
| Exp. 2 | Found Target | 0.36 (0.23) | 10.4 (5.0) |  |  |  |
|  | False Alarm | 0.01 (0.01) | 28.2 (27.9) |  |  |  |
|  | Selected Later | 0.19 (0.22) | 10.4 (2.7) | 1876 (1872) |  |  |
|  | Selected Later, Incorrectly | 0.01 (0.01) | 17.1 (20.1) | 1802 (2637) |  |  |
|  | Liking Procedure  (CB trial) | 0.19 (0.11) | 9.5 (2.1) | 595 (91) | 0.08 (0.20) | -0.06 (0.23) |
|  | Liking Procedure on Catch Trial | 0.24 (0.01) | 17.0 (2.7) | 742 (195) | 0.07 (0.15) | 0.00 (0.10) |


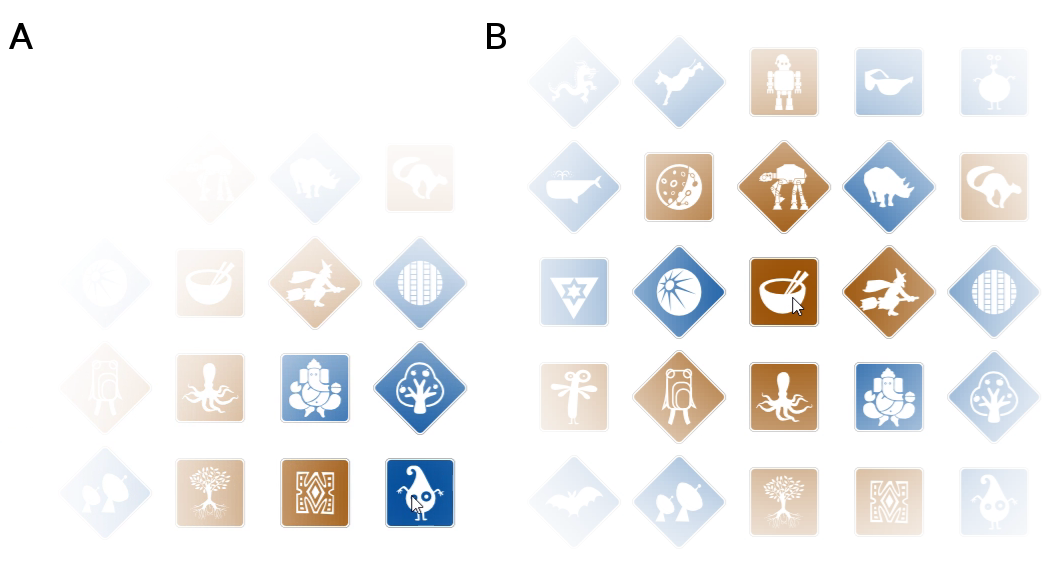


Figure S1. Example of the mask used when mouse cursor (Experiment 1) or gaze (Experiment 2) were at the corner stimuli (panel A) or at the center (panel B). In both cases the mask shown has the maximal size. When cursor or gaze stayed at the same position, the mask began to shrink (see details in text).


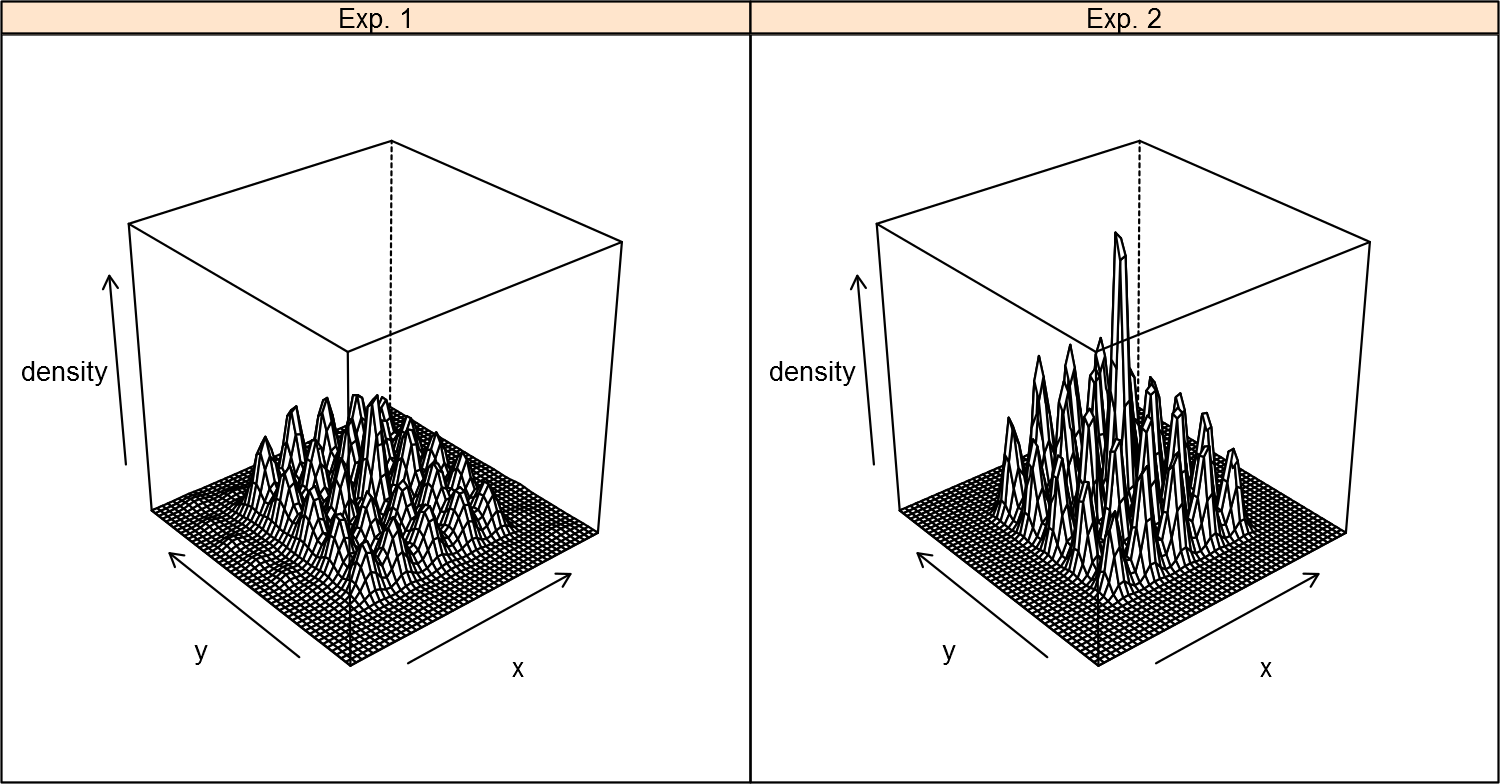


Figure S2. Alternative presentation of fixation distribution in the two experiments. The data is the same as in Figure 2. The plots show the probability density of fixations with different coordinates (x and y). Higher peaks indicate that the fixation at a given location were more probable. Both plots are normalized to sum to one, so that higher peaks in Exp. 2 compared to Exp. 1 mean that the between-peak “valleys” are lower.
